# LAUNCHING THE PORCHLIGHT PROJECT

**DOI:** 10.1093/geroni/igad104.1087

**Published:** 2023-12-21

**Authors:** Joseph Gaugler, Elizabeth Albers, Katie Louwagie, Noelle Fields, Eric Jutkowitz, Manka Nkimbeng, Tetyana Shippee, David Roth

**Affiliations:** University of Minnesota, Minneapolis, Minnesota, United States; University of Minnesota School of Public Health, Minneapolis, Minnesota, United States; University of Minnesota, Minneapolis, Minnesota, United States; The University of Texas at Arlington, Arlington, Texas, United States; Brown University, Providence, Rhode Island, United States; University of Minnesota, Minneapolis, Minnesota, United States; University of Minnesota, Minneapolis, Minnesota, United States; Johns Hopkins University, Baltimore, Maryland, United States

## Abstract

The Porchlight Project (PLP) is a 5-year study designed to evaluate the real-world efficacy of dementia capable training and respite delivered by volunteers for older people living in the community throughout Minnesota and in select regions of North Dakota. Volunteer regions managed by the Lutheran Social Services of Minnesota (LSS-MN) throughout Minnesota and North Dakota were randomly assigned to receive the dementia training and volunteer support of PLP or the usual care control condition with standard volunteer training. The purpose of this presentation is to describe the process involved in establishing the PLP within LSS-MN’s routine programming for volunteers and embedding key outcome measures within LSS-MN’s standard data collection process for volunteers, their clients, and their clients’ caregivers. Various challenges to PLP integration included: human subjects research categorization for an evaluation such as the PLP; achieving survey response rate goals; and establishing a baseline data collection interval prior to volunteer training completion. Strategies to proactively address these challenges to promote successful embedding of the program include creation of a PLP coordinator position within LSS-MN, who is responsible for the day-to-day programming of the project, as well as the development of a robust, yet flexible protocol. The flexibility allows for navigating LSS-MN programmatic activities, such as changes to the LSS-MN internal database, fluctuation in staffing and/or volunteers, revisions to survey follow-up procedures and correspondence, and consideration for those who will not complete training (e.g. non-English speakers, etc.). This presentation will also feature baseline descriptive data from the evaluation.

